# Resveratrol Induces Proteasomal Degradation of PTPN1 to Enhance Cisplatin Sensitivity in Epstein–Barr Virus-Associated Malignancies

**DOI:** 10.3390/ph19040603

**Published:** 2026-04-09

**Authors:** Na Liu, Yueshuo Li, Min Tang, Ya Cao, Li Shang, Feng Shi

**Affiliations:** 1 Key Laboratory of Carcinogenesis and Cancer Invasion of Chinese Ministry of Education, Xiangya Hospital, Central South University, Changsha 410078, China; liu_na@csu.edu.cn (N.L.); mgxxwn@csu.edu.cn (Y.L.); tangmin@csu.edu.cn (M.T.); ycao98@csu.edu.cn (Y.C.); 2 Department of Pathology, National Clinical Research Center for Geriatric Diseases (Xiangya Hospital), Central South University, Changsha 410078, China; 3 Key Laboratory of Carcinogenesis of National Health Commission, Cancer Research Institute, Xiangya School of Basic Medical Sciences, Central South University, Changsha 410078, China; 4 Molecular Imaging Research Center, Central South University, Changsha 410078, China

**Keywords:** resveratrol, PTPN1, Epstein–Barr virus, cisplatin

## Abstract

**Background/Objectives**: EBV is an oncogenic virus linked to NPC and GC, driving cisplatin resistance. Resveratrol has anticancer activity, but its targets and mechanisms against EBV-positive cancers remain unclear. **Methods**: We assessed resveratrol’s cytotoxicity in EBV-positive cells via functional assays, identified targets by chemical similarity search and molecular docking, and validated PTPN1 via in vitro experiments and nude mouse xenograft models. **Results**: Resveratrol inhibited EBV-positive cell viability in a time- and concentration- dependent manner, with IC50 values ranging from 35.85 to 145.7 μM across different cell lines at 24–72 h. Apoptosis rates increased by approximately 2- to 4-fold after 80 μM resveratrol treatment for 24 h. Resveratrol directly targeted PTPN1 (docking score = −4.89) and promoted its degradation via the proteasome pathway, as MG132 reversed this effect. Notably, resveratrol synergized with cisplatin (combination index < 1) to reverse cisplatin resistance in both in vitro and in vivo models. Furthermore, resveratrol induced EBV lytic reactivation through ROS production, as evidenced by the increased expression of BZLF1, BMRF1, and BALF2, which was attenuated by the ROS scavenger NAC. **Conclusions**: Our findings identify PTPN1 as a direct anticancer target of resveratrol in EBV-positive cancers. Resveratrol enhances the therapeutic efficacy of cisplatin via PTPN1 proteasomal degradation and induces EBV lytic reactivation through ROS accumulation. These findings provide a mechanistic basis for the development of novel combination therapies targeting EBV-associated malignancies.

## 1. Introduction

Epstein–Barr virus (EBV) belongs to the γ-herpesvirus family and is the first identified human oncogenic virus, infecting over 95% of the global adult population and establishing persistent latent infection in the host [1,2,3]. There are two distinct phases in the life cycle of EBV, latency and lytic replication [2]. During the latency phase, only a limited number of viral genes are expressed, such as latent member protein 1 (LMP1), EBV Nuclear Antigens (EBNAs) and EBV-Encoded RNAs (EBERs) [4]. The transition to the lytic phase allows the virus to replicate its genome via genes like BZLF1 (Zta), BRLF1 (Rta), and BMRF1, ultimately producing infectious progeny [2]. LMP1 is a key oncoprotein encoded by EBV and plays critical roles in latent infection [5]. It is closely associated with the pathogenesis and progression of EBV-associated tumors, serving as a prognostic indicator in nasopharyngeal carcinoma (NPC) [4,6]. Approximately 9% of gastric carcinoma (GC) patients are EBV-positive, exhibiting distinct molecular phenotypes and clinical features, such as global epigenetic methylation and a reactive anti-tumor microenvironment [4,7]. Both NPC and GC contribute significantly to global cancer-related morbidity, and EBV infection often correlates with unfavorable outcomes, underscoring the urgent need for novel therapeutic strategies.

Chemotherapy remains a cornerstone in the treatment of NPC and GC. However, acquired resistance severely compromises patient outcomes [8,9]. EBV plays a pivotal role in the acquisition and maintenance of chemotherapy resistance. For instance, EBV infection-induced GPX4 promotes cisplatin resistance and tumor progression in NPC [10]. Exosomal circPARD3 enhances stemness and cisplatin resistance induced by EBV-miR-BART4 in NPC side population cells via the miR-579-3p/SIRT1/SSRP1 pathway [11]. Our previous studies demonstrated that EBV-LMP1 enhances cisplatin resistance through multiple signaling pathways, including the regulation of the mitochondrial permeability transition pore via ANT1 binding and modulation of Drp1 through AMPK and cyclin B1/Cdk1 axes [12,13]. These findings highlight the need for agents that can overcome EBV-mediated chemoresistance.

Resveratrol (3,4′,5-trihydroxy-trans-stilbene), a naturally occurring non-flavonoid polyphenol compound, is a stilbene extracted from Polygonum cuspidatum, Cassia seeds, and grapes [14]. It exists as cis and trans isomers, with the trans isomer being more stable, biologically active, and responsible for its anticancer benefits [15]. Resveratrol exhibits favorable safety profiles, multitargeting capabilities, and the potential to modulate chemosensitivity, making it an attractive candidate for combination therapy. Extensive research has demonstrated its anticancer potential across various tumor types, including cervical cancer, breast cancer, non-small cell lung cancer, gastric cancer, prostate cancer, ovarian cancer, multiple myeloma, and triple-negative breast cancer [14,16,17,18,19,20,21,22]. For example, resveratrol activates endoplasmic reticulum stress-induced senescence in breast cancer and lung cancer cells through the SIRT1/p38MAPK and NO/DLC1 pathways [23]. It modulates the chemosensitivity of colorectal cancer to fluorouracil via the β1-Integrin/HIF-1α axis [24]. Resveratrol suppresses the metastasis of triple-negative breast cancer cells by up-regulating the expression of CDH1 and CDKN1A [20]. Moreover, network pharmacology and molecular docking analyses have revealed that resveratrol can directly target a diverse array of genes, including MAPK3, VGFR3, AKR1C3, AKT1, and TP53, to exert its tumor-suppressive effects [25,26,27,28]. Despite numerous studies validating the anticancer activity of resveratrol, the impact of resveratrol on EBV-positive tumor cells remains unclear.

Protein tyrosine phosphatase non-receptor type 1 (PTPN1) is a ubiquitously expressed phosphatase implicated in various physiological and pathological processes [29]. PTPN1 is up-regulated in multiple cancers, such as melanoma, pancreatic cancer, and glioma, and promotes tumor cell proliferation, migration and invasion [30,31,32]. Notably, the inhibition of PTPN1 has been shown to enhance the sensitivity of cancer cells to chemotherapeutic agents, including paclitaxel and cisplatin [33,34]. Moreover, PTPN1 serves as an intracellular checkpoint in T cells, and its increased expression limits anti-tumor immunity [35]. These findings underscore the potential of PTPN1 as a promising therapeutic target, particularly in overcoming drug resistance.

In this study, we employed an integrated strategy combining target prediction, molecular docking, and experimental validation to investigate the anti-tumor mechanisms of resveratrol in EBV-positive NPC and GC cells. Our primary objectives were to determine whether PTPN1 acts as a direct molecular target of resveratrol, to clarify how resveratrol sensitizes cancer cells to cisplatin, and to explore its role in inducing EBV lytic reactivation. Through these investigations, we aim to establish a mechanistic basis for the development of novel combination therapies targeting EBV-associated malignancies.

## 2. Results

### 2.1. Resveratrol Significantly Inhibits the Viability of EBV/LMP1-Positive Cell Lines

**Figure 1:**
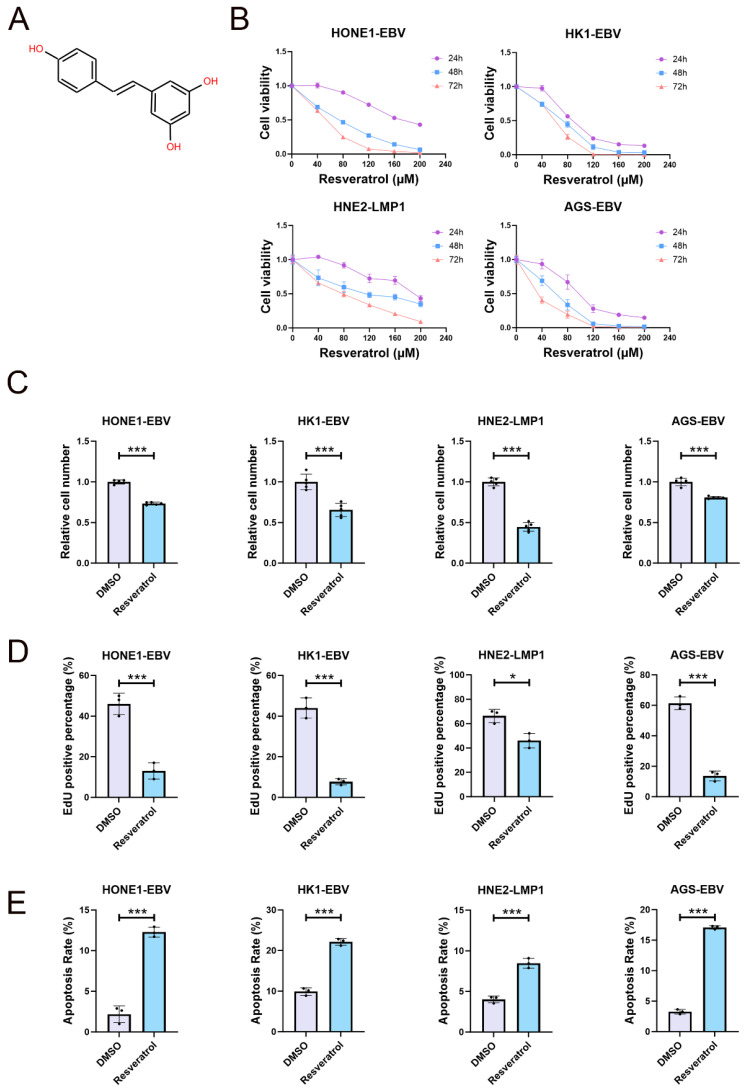
Resveratrol inhibits the viability of EBV/LMP1-positive cell lines. (**A**) The chemical structure of resveratrol, a small molecule compound known for its potential anticancer properties. (**B**) HONE1-EBV, HK1-EBV, HNE2-LMP1, and AGS-EBV cells were treated with various concentrations of resveratrol (0 µM as a control, 40 µM, 80 µM, 120 µM, 160 µM, and 200 µM) for durations of 24 h, 48 h, and 72 h. The effect of resveratrol on cell viability was assessed using the CCK-8 assay, with each treatment conducted in quintuplicate (n = 5). (**C**) HONE1-EBV, HK1-EBV, HNE2-LMP1, and AGS-EBV cells were treated with 80 μM resveratrol for durations of 24 h. The number of cells was assessed by using a Lionheart LX automated microscope (n = 5; ***, *p*  <  0.001). (**D**) EdU staining was performed to assess the S-phase cell cycle progression in cells treated with DMSO and resveratrol (80 µM, 24 h). Hoechst 33342 staining was used to visualize nuclei (n = 3; *, *p*  <  0.05; ***, *p*  <  0.001). (**E**) HONE1-EBV, HK1-EBV, HNE2-LMP1, and AGS-EBV cells were treated with 80 μM resveratrol for durations of 24 h. A flow cytometry analysis of cell death (n = 3; ***, *p*  <  0.001).

We commenced our investigation by examining the impact of resveratrol (whose structure is depicted in Figure 1A) on the viability of EBV-positive NPC and GC cells by using the CCK-8 assay. We treated HONE1-EBV, HK1-EBV, HNE2-LMP1, and AGS-EBV cell lines with varying concentrations of resveratrol over different durations. Our findings revealed that resveratrol significantly diminished cell viability in a concentration- and time-dependent manner (Figure 1B). IC50 at different times is shown in Table 1. Notably, the cell counts dramatically declined following treatment with resveratrol (Figure 1C). The EdU assay further confirmed that resveratrol suppressed the proliferation of cancer cells, evidenced by a reduction in EdU-positive cells (Figure 1D and Supplementary Figure S1A).

Subsequently, we evaluated the effects of resveratrol on the apoptosis of these cancer cells. We measured the proportion of apoptotic cells after treatment with specified concentrations of resveratrol. Flow cytometry analysis indicated that the apoptosis rates in HONE1-EBV, HK1-EBV, HNE2-LMP1, and AGS-EBV cells pre-treated with resveratrol were notably higher compared to the control groups (Figure 1E and Supplementary Figure S1B). These outcomes suggest that resveratrol exerts a substantial cytotoxic effect against EBV-positive cell lines.

It has been reported that resveratrol can cause cellular senescence and various types of cell death by evoking ROS generation [36,37]. In this study, we confirmed that resveratrol could stimulate ROS production via flow cytometry analysis (Supplementary Figure S2A). However, the rescue of cell proliferation by the addition of the ROS scavenger acetylcysteine (NAC) was not observed (Supplementary Figure S2B), implying that resveratrol’s cytotoxic mechanisms may operate through alternative pathways.

### 2.2. PTPN1 Is a Promising Target of Resveratrol

**Figure 2:**
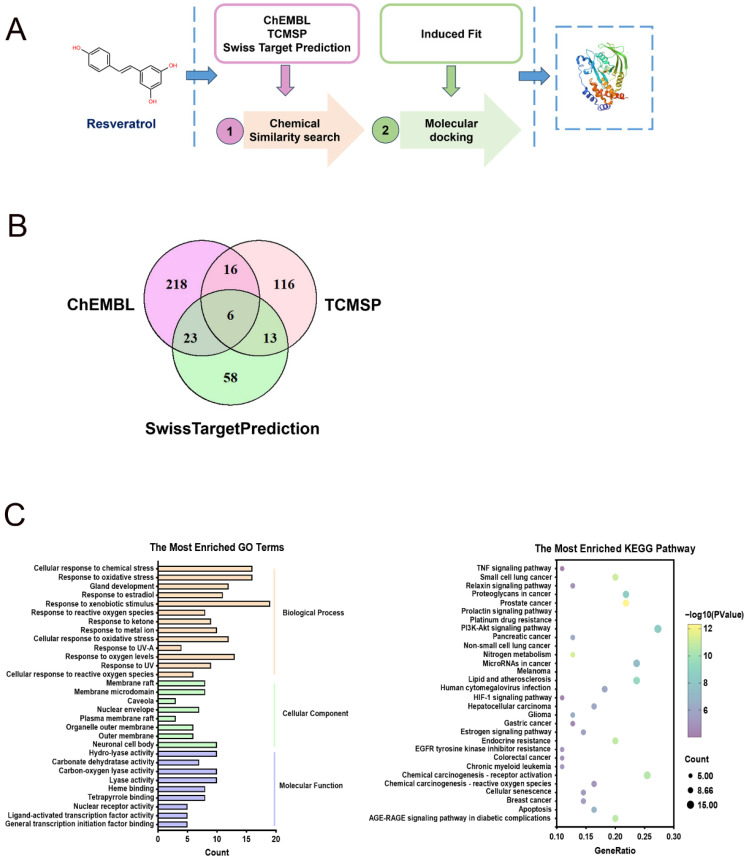
The combinatorial target prediction strategy identifies potential targets of resveratrol. (**A**) The stepwise combinatorial target prediction strategy was used to screen potential target proteins of resveratrol. (**B**) Overlapping target proteins predicted by various target prediction platforms. (**C**) The top enriched GO terms and KEGG pathway analysis to validate the target protein effects.

To elucidate the mechanisms of resveratrol effect on cancer cells, we adopted an integrated approach for target prediction, which could systematically identify potential targets of resveratrol (Figure 2A). Our initial screening relied on chemical structure similarities to compile a list of candidate proteins, by utilizing three ligand-based prediction platforms—ChEMBL, TCMSP, and Swiss Target Prediction. We collated the predictions to derive overlapping targets and produced a Venn diagram which revealed 58 target proteins common to at least two platforms (Figure 2B and Supplementary Table S1). These proteins exhibit a strong association with key biological processes linked to inflammation, reactive oxygen species (ROS), and cancer. This alignment was substantiated by the predominant KEGG pathways and GO terms, such as the TNF signaling pathway, PI3K-AKT signaling pathway, cellular senescence, apoptosis, general transcription initiation factor binding, cellular response to reactive oxygen species, and cellular response to chemical stress (Figure 2C).

**Figure 3:**
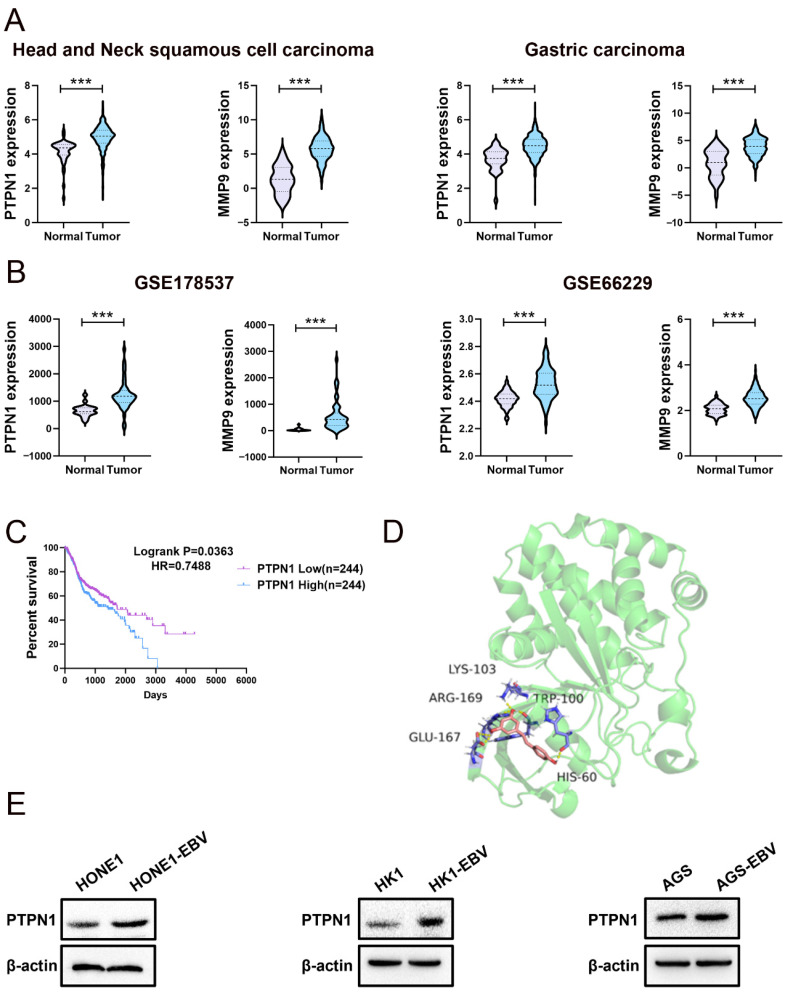
PTPN1 is a promising target of resveratrol. (**A**,**B**) Gene expression analysis utilizing TCGA (TCGA-HNSCC dataset: Normal = 44, Tumor = 520; TCGA-GC dataset: Normal = 36, Tumor = 414) and GEO databases (GSE178537: Head and neck squamous cell carcinoma samples, Normal = 21, Tumor = 44; GSE66229: Gastric cancer samples, Normal = 100, Tumor = 300; ***, *p*  <  0.001). (**C**) A Kaplan–Meier curve showing the overall survival probability of HNSCC patients from the TCGA database with low or high mRNA levels of PTPN1. (**D**) The optimal structural conformation of PTPN1 (Green and Purple) and the ligand resveratrol (Pink). Docking score= −4.89. (**E**) The protein expression of PTPN1 in HONE1/HONE1-EBV, HK1/HK1-EBV, and AGS/AGS-EBV cell lines.

Subsequently, we shortlisted six target proteins consistently predicted across all platforms, including protein tyrosine phosphatase non-receptor type 1 (PTPN1), matrix metallopeptidase 9 (MMP9), matrix metallopeptidase 2 (MMP2), BCL2 apoptosis regulator (BCL2), carbonic anhydrase 2 (CA2) and prostaglandin-endoperoxide synthase 2 (PTGS2) (Figure 2B and Supplementary Table S1). To investigate the expression levels of these six genes in cancer tissues, we conducted a comprehensive analysis utilizing data from both the TCGA database and GEO datasets. Specifically, within the GEO repository, we examined head and neck squamous cell carcinoma (HNSCC) samples from GSE178537 and gastric cancer samples from GSE66229. An analysis of these datasets revealed a significant overexpression of PTPN1 and MMP9 in these cancer types (Figure 3A,B and Supplementary Figure S3A,B). Tumor samples from the TCGA-HNSCC dataset were stratified into two groups based on the median expression levels of the PTPN1 and MMP9 genes. Subsequently, Kaplan–Meier survival analyses revealed a significant association between PTPN1 expression and overall survival in HNSCC patients. Specifically, patients with low PTPN1 expression demonstrated improved survival outcomes compared to those with high expression (Figure 3C). In contrast, MMP9 gene expression levels did not exhibit a statistically significant correlation with overall survival in HNSCC patients (Supplementary Figure S3C). COX regression analysis indicated that high PTPN1 expression emerged as an independent prognostic factor of HNSCC patients (Table 2). It has been demonstrated that PTPN1 plays crucial roles in the development of tumors, such as promoting cell proliferation and the migration and invasion of tumor cells. Given the tumorigenic role PTPN1 plays across various tumor types, our research centered on PTPN1.

Molecular docking analyses were conducted to evaluate the affinity and bonding interactions between PTPN1 and resveratrol. It was revealed that hydrogen bonding plays a crucial role in the binding of resveratrol to PTPN1, with a docking score of −4.89, which signifies a robust interaction (Figure 3D and Supplementary Figure S3D). Western blot assay revealed that PTPN1 was highly expressed in EBV-positive NPC and GC cells (Figure 3E). These data suggested that PTPN1 is a reliable target of resveratrol.

### 2.3. Resveratrol Promotes PTPN1 Degradation via the Proteasome Pathway

**Figure 4:**
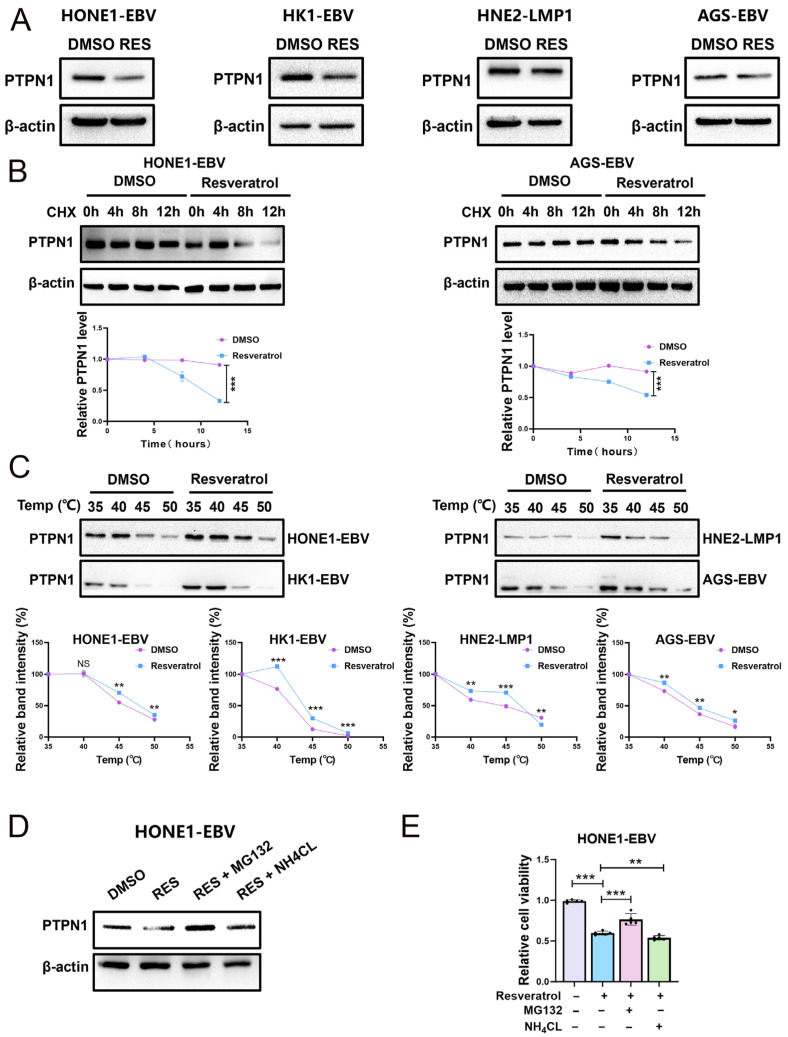
Resveratrol exerts anticancer activity by promoting the protein degradation of PTPN1. (**A**) EBV/LMP1-positive NPC and GC cells treated with resveratrol (80 µM) for 24 h were analyzed by Western blotting to detect PTPN1 expression levels (RES: resveratrol). (**B**) HONE1-EBV and AGS-EBV cells were treated with DMSO or resveratrol (80 µM, 12 h), followed by 20 μg/mL CHX to inhibit protein synthesis. Cells were collected at specified time points and subjected to Western blotting using anti-PTPN1 and anti-β-actin antibodies. The protein levels of PTPN1 were quantified by normalizing to β-actin expression (***, *p*  <  0.001). (**C**) CETSA was used to evaluate the binding between resveratrol (80 µM) and PTPN1 at thermodynamic levels. The expression of PTPN1 was detected by Western blotting (NS, >0.05; *, *p*  <  0.05; **, *p*  <  0.01; ***, *p*  <  0.001). (**D**) HONE1-EBV cells were treated with resveratrol (80 µM), resveratrol combined with the proteasome inhibitor MG132 (10 µM), and resveratrol combined with the autophagy–lysosome inhibitor NH4CL (5 mM) for 24 h. The protein level of PTPN1 was then measured by Western blotting. (**E**) HONE1-EBV cells were treated with resveratrol (80 µM), resveratrol combined with the proteasome inhibitor MG132 (10 µM), and resveratrol combined with the autophagy–lysosome inhibitor NH4CL (5 mM) for 24 h. Cell viability was assessed by using the CCK-8 assay (n = 5; **, *p*  <  0.01; ***, *p*  <  0.001).

As shown in Figure 4A, the expression of the PTPN1 protein was obviously down-regulated in cells treated with resveratrol (Figure 4A). Next, we hypothesized that resveratrol could impair the stability of PTPN1. Following the inhibition of protein synthesis with cycloheximide (CHX), total protein extracts were collected at designated time points. The data illustrated that resveratrol treatment notably reduced the half-life of PTPN1 (Figure 4B). To further confirm the direct interaction between resveratrol and PTPN1, we conducted a cellular thermal shift assay (CETSA) in NPC and GC cells. The results demonstrated that the thermal stability of PTPN1 was enhanced following resveratrol treatment (Figure 4C), implying the direct engagement of resveratrol with PTPN1. We further observed that the degradation of PTPN1 can be reversed by the proteasome inhibitor MG132 but not the autophagy inhibitor NH4CL (Figure 4D), suggesting that resveratrol decreases the stability of PTPN1 by promoting its degradation via the proteasome pathway. Furthermore, the addition of MG132 can reverse the reduction in cell viability caused by resveratrol (Figure 4E). Thus, resveratrol exerts its anticancer activity by promoting the degradation of PTPN1.

PTPN1 expression has been closely linked with the presence of tumor-infiltrating immune cells, as well as the expression of genes associated with immune checkpoint pathways across various cancers [33]. Furthermore, PTPN1 serves as a pivotal mediator of inflammation, with its genetic ablation in either tumor or immune cells enhancing anti-tumor immunity [35,38]. The mRNA expression levels of type I interferon and interleukin-6 were increased in NPC and GC cells treated with resveratrol (Supplementary Figure S4A). To a certain extent, resveratrol exerts its anti-tumor immune function by inhibiting the expression of PTPN1. Consequently, PTPN1 has emerged as a prime candidate for in-depth investigation as a potential target of resveratrol.

### 2.4. Resveratrol Enhances the Cytotoxicity of Cisplatin to EBV-Positive Cancer Cells

**Figure 5:**
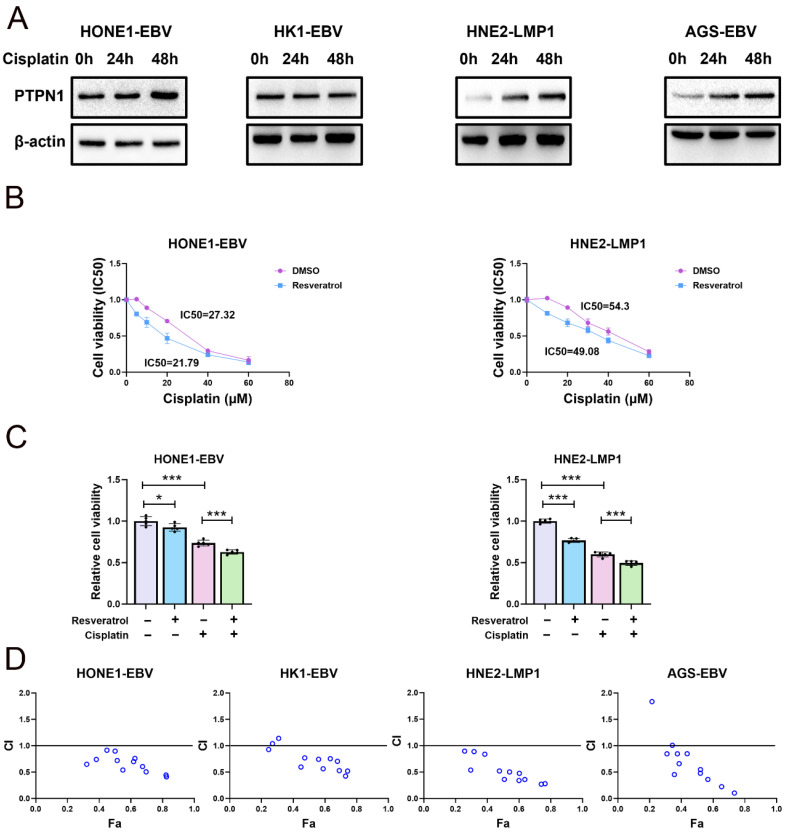
Resveratrol enhances the cytotoxicity of cisplatin. (**A**) HONE1-EBV, HK1-EBV, HNE2-LMP1 and AGS-EBV cells treated with cisplatin (10 μM) for 0 h, 24 h, and 48 h. Cells were collected to detect PTPN1 expression levels by Western blotting. (**B**) HONE1-EBV and HNE2-LMP1 cells were treated with cisplatin (0 µM, 10 µM, 20 µM, 30 µM, 40 µM, and 60 µM) alone or cisplatin (0 µM, 10 µM, 20 µM, 30 µM, 40 µM, and 60 µM) in combination with resveratrol (80 µM) for 24 h. The IC50 value of cisplatin in HONE1-EBV and HNE2-LMP1 cells. (**C**) HONE1-EBV and HNE2-LMP1 cells were treated with cisplatin (10 μM) alone or cisplatin (10 μM) in combination with resveratrol (80 µM) for 24 h. Cell viability was measured by CCK-8 assay (n = 5; *, *p*  <  0.05; ***, *p*  <  0.001). (**D**) The combination index of cisplatin and resveratrol (note: CI  <  1 indicates synergism, CI  =  1 indicates an additive effect, and CI  >  1 indicates antagonism).

Cisplatin-based chemoradiotherapy is a standard therapeutic approach for NPC and GC. Yet the varying degrees of patient sensitivity to cisplatin, coupled with its adverse effects, continue to pose significant challenges in managing this cancer. Hence, it is critically important to develop novel treatment strategies to improve cisplatin sensitivity. PTPN1 expression is significantly increased in cisplatin-resistant cells [34]. Silencing PTPN1 enhances the sensitivity to cisplatin in cisplatin-resistant colorectal, cervical, and gastric cancer cells [34,39]. EBV/LMP1-positive cancer cells were revealed as being more resistant to cisplatin than EBV/LMP1-negative cells. This chemoresistance may be associated with the up-regulation of PTPN1 expression mediated by EBV. It has been demonstrated that cisplatin could bind to PTPN1 and inhibit its enzymatic activity [40]. This raises the question whether the combination of resveratrol and cisplatin could lead to enhanced anticancer activity by targeting PTPN1. As shown in Figure 5A, PTPN1 expression was elevated after cisplatin treatment (Figure 5A). We wondered whether resveratrol could mitigate the up-regulatory effect on PTPN1 induced by cisplatin. To test this, we assessed the IC50 values of cisplatin in HONE1-EBV and HNE2-LMP1 cells with and without resveratrol treatment. IC50 was significantly lower in the resveratrol treatment group (Figure 5B). Additionally, we evaluated the cell viability of EBV/LMP1-positive cancer cells treated with cisplatin either alone or in combination with resveratrol. In EBV/LMP1-positive cells, the combination of resveratrol and cisplatin significantly decreased cell viability compared to cisplatin treatment alone (Figure 5C). To ascertain whether the combined effect of cisplatin and resveratrol exceeded the sum of their individual effects (synergistic interaction), we computed combination indexes (CIs) from dose–response data. The CI values for NPC cells treated with the cisplatin–resveratrol combination were less than 1, suggesting a synergistic effect (Figure 5D).

**Figure 6:**
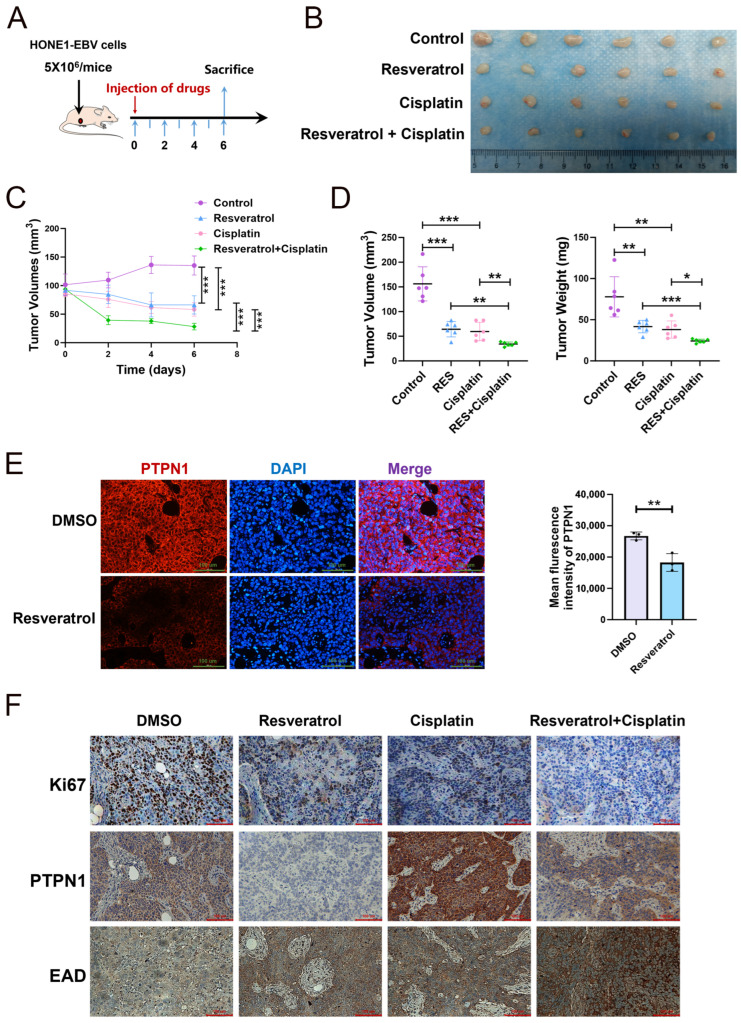
Resveratrol enhances the cytotoxicity of cisplatin in the NPC xenograft model. (**A**) The overall diagram of the study design. (**B**) Representative images of xenografts from different treatment groups. (**C**) Changes in tumor volume across days with different treatments (n = 5; ***, *p*  <  0.001). (**D**) The final tumor volume and tumor weight of HONE1-EBV-derived xenograft tumors with various treatments (n = 5; *, *p*  <  0.05; **, *p*  <  0.01; ***, *p*  <  0.001; RES: resveratrol). (**E**) Representative immunohistochemical images showing PTPN1 staining in xenograft tumors (scale bar, 100 μm) (n = 3; **, *p*  <  0.01). (**F**) Tumor sections were subjected to immunohistochemistry detection for Ki67, PTPN1 and EAD (scale bar, 100 μm).

To delve deeper into the anti-tumor effects of resveratrol, we treated nude mice bearing HONE1-EBV xenografts with either resveratrol, cisplatin, or the combination of resveratrol and cisplatin (Figure 6A). As the duration of treatment increased, the tumor volume in mice receiving a combination of cisplatin and resveratrol was significantly smaller compared to those treated with either cisplatin or resveratrol alone (Figure 6B,C). The final tumor volume and weight also demonstrated a significant tumor suppression effect in the combination group (Figure 6D). And the treatment of resveratrol or/and cisplatin did not significantly affect body weight in mice (Supplementary Figure S4B). Thus, these findings suggest that resveratrol enhances the sensitivity of HONE1-EBV xenografts to cisplatin. Further investigation showed that PTPN1 expression was significantly reduced in tissues from the resveratrol treatment group (Figure 6E,F). Furthermore, the Ki67 assay demonstrated that treatment with resveratrol or/and cisplatin effectively suppressed cell proliferation in xenografts (Figure 6F). The PTPN1 protein expression level was higher in the cisplatin treatment group, i.e., tumor cells enhanced their resistance to cisplatin by up-regulating PTPN1 expression (Figure 6F). Meanwhile, resveratrol can enhance the sensitivity to cisplatin by inhibiting PTPN1 expression (Figure 6F).

### 2.5. Resveratrol Induces EBV into Lytic Phase in NPC and GC Cells

It has been reported that radiation and cisplatin treatment could up-regulate the expression of EBV lytic genes, BRLF1 and BZLF1, and induce EBV lytic reactivation [41]. Chemotherapeutic agents such as cisplatin, 5-fluorouracil, and paclitaxel have also been shown to induce EBV lytic reactivation [42,43]. The reactivation of EBV lytic genes by cisplatin in combination with DNA methylation inhibitors may represent a novel therapeutic approach for EBV-associated gastric cancer [44]. Valproic acid effectively enhances the ability of various chemotherapeutic agents to activate EBV lytic genes, increasing the cytotoxicity of these agents in an EBV-dependent manner [45].

**Figure 7:**
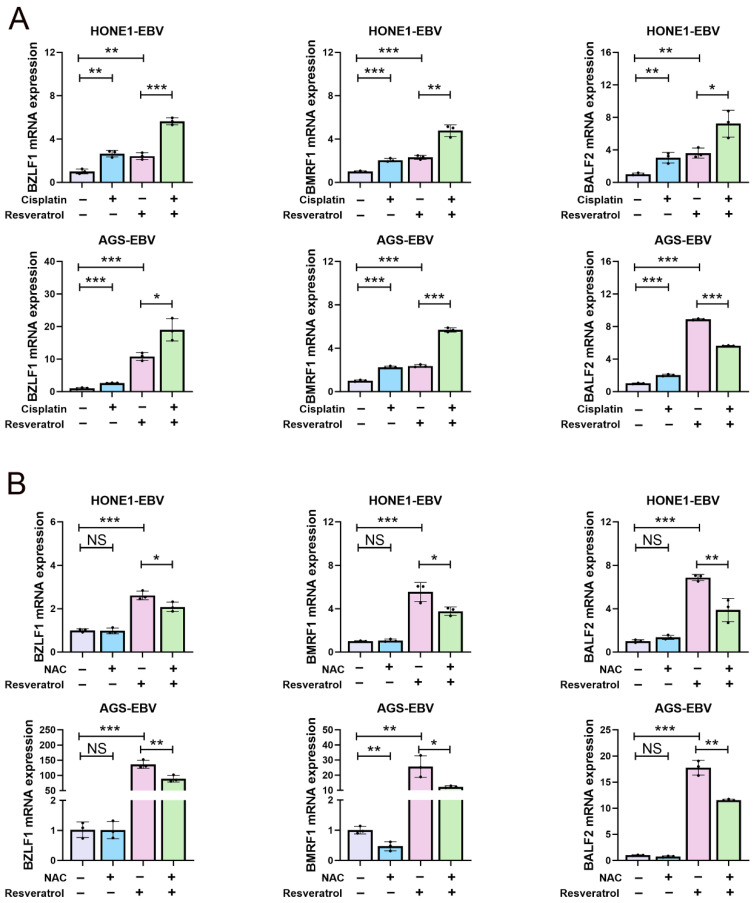
Resveratrol induces EBV into the lytic phase. (**A**) EBV-positive cells were treated with cisplatin (10 μM), resveratrol (80 µM), or a combination of both cisplatin (10 μM) and resveratrol (80 µM) for 24 h. mRNA expression levels were assessed by using qPCR (n = 3; *, *p*  <  0.05; **, *p*  <  0.01; ***, *p*  <  0.001). (**B**) EBV-positive cells were treated with NAC (5 mM), resveratrol (80 µM), or a combination of both NAC (5 mM) and resveratrol (80 µM) for 24 h. mRNA expression levels were assessed by using qPCR (n = 3; NS, >0.05; *, *p*  <  0.05; **, *p*  <  0.01; ***, *p*  <  0.001).

As shown in Figure 7A, both cisplatin and resveratrol treatment induced the mRNA expression of EBV lytic genes (BZLF1, BMRF1 and BALF2), which are enhanced by the combination treatment (Figure 7A). This suggests that both resveratrol and cisplatin may induce EBV lytic reactivation, and the effect is significantly stronger in combination. In the tissues of HONE1-EBV xenografts, EAD, encoded by EBV lytic gene BMRF1, was highly expressed in the combination treatment group (Figure 6A,B,F). Thus, the combination of resveratrol and cisplatin may inhibit cell viability by promoting EBV lytic-induced cell rupture.

Furthermore, we established that resveratrol is able to induce the production of ROS (Supplementary Figure S2A). It has been previously demonstrated that the enhanced production of ROS facilitates the lytic reactivation of EBV [46]. This raises the question whether ROS plays a pivotal role in resveratrol-induced EBV lytic gene expression. As shown in Figure 7B, the mRNA expression of the BZLF1, BMRF1, and BALF2 genes was significantly reduced in the presence of the ROS inhibitor NAC (Figure 7B), suggesting that resveratrol indeed triggers the lytic reactivation of EBV by inducing ROS production.

## 3. Discussion

**Figure 8:**
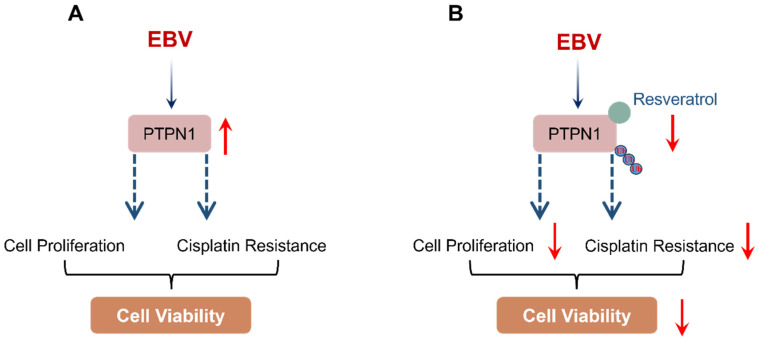
A schematic model illustrating the anticancer mechanism of resveratrol in EBV-positive NPC and GC cells. (**A**) PTPN1 expression is markedly up-regulated in EBV-positive tumors. (**B**) As a bioactive small molecule, resveratrol directly interacts with PTPN1 and induces its degradation via the proteasome pathway. By down-regulating PTPN1, resveratrol inhibits cell viability and enhances the therapeutic efficacy of cisplatin, thereby exerting potent anticancer effects on EBV-positive NPC and GC cells.

Traditional Chinese medicine has shown promising anticancer efficacy and considerable therapeutic potential in the treatment of cancer. However, the lack of clarity regarding the molecular mechanisms underlying these effects has significantly impeded its clinical deployment in oncology. Enhancing our understanding of the molecular actions through which these natural product preparations exert anti-tumor effects is critical for the development of these compounds into effective anticancer agents. Among them, resveratrol has a favorable safety profile and exhibits broad anticancer activity across multiple tumor types [14,16,17,18,19,20,21,22]. Resveratrol possesses multilevel, multitarget, and coordinated intervention effects, making it an ideal candidate for the inhibition of tumor progression [47]. While the anticancer properties of resveratrol are increasingly recognized, its roles in EBV-positive NPC and GC remain poorly understood. Investigating the molecular pathways modulated by resveratrol could pave the way for the creation of new anticancer drugs. In this study, we demonstrated the substantial anticancer activity of resveratrol against EBV-positive NPC and GC cells. Notably, using a combined approach of target prediction and subsequent validation, we identified PTPN1 as a potential direct target of resveratrol, underscoring its role in mediating resveratrol-induced anticancer effects (Figure 8).

PTPN1 is a ubiquitously expressed protein involved in a variety of physiological and pathological processes [35]. Elevated levels of PTPN1 have been observed in breast cancer, gastric cancer, and glioma, which results in promoting tumor growth [32,48]. Numerous research findings have validated that the inhibition of PTPN1 activity represents a promising novel approach for tumor treatment and metastatic diseases [33,38,49]. For instance, PTPN1-deficient HER2(+) xenografts exhibit enhanced hypoxia, increased necrosis, and reduced growth [50]. Similarly, OA-Br-1, a novel PTPN1 inhibitor, exerts anti-breast cancer effects by suppressing the PTP1B/PI3K/AKT signaling pathway [51]. In line with these findings, our data suggested that resveratrol could impair the cell viability of EBV-positive NPC and GC cells by promoting PTPN1 degradation.

Cisplatin-based chemotherapy has been shown to substantially extend the life expectancy of patients with NPC and is therefore advocated as the preferred treatment strategy. However, the development of therapeutic resistance often leads to treatment failure, imposing limitations on survival improvements [8,9]. Accumulating evidence suggests that resveratrol, a natural compound with a favorable safety profile, may enhance the anticancer efficacy of cisplatin. It has been reported that the combination of cisplatin and resveratrol increases apoptosis and autophagy under oxidative stress [52]. This combination therapy also inhibits metastasis and induces apoptosis and senescence by targeting the P38/P53 and P16/P21 pathways [53]. Moreover, resveratrol enhances the cytotoxic effects of cisplatin by activating P38 and inhibiting AKT [14]. By binding with CARM1, resveratrol increases the methyltransferase activity of CARM1, which further enhances the anticancer effects of cisplatin [54]. Additionally, resveratrol inhibits the production of reduced glutathione by decreasing the expression of the glutamine transporter protein ASCT2, which disrupts the intracellular redox state and further increases the sensitivity of cancer cells to cisplatin [55]. These studies collectively elucidate the potential of combining resveratrol with cisplatin for cancer therapy. In our study, we found that PTPN1 levels were elevated in EBV-positive cells and gradually increased with prolonged cisplatin treatment. Decreasing PTPN1 expression via resveratrol enhanced the sensitivity of these cells to cisplatin, and the combined treatment exhibited a synergistic effect in overcoming resistance in EBV-positive NPC and GC cells. These findings suggest a significant connection between PTPN1 and cisplatin resistance and indicate that the combination of resveratrol with cisplatin may represent a novel therapeutic strategy to overcome cisplatin resistance in EBV-positive tumors.

EBV infection consists of two phases, latency and lytic replication [56]. During the lytic phase, EBV typically results in cell death when it replicates [2]. The induction of lytic replication has been considered as a novel strategy for the treatment of EBV-positive tumors [57,58]. This strategy leverages the presence of EBV to exert a selective cytotoxic effect on EBV-associated malignancies. For instance, thymidine kinase, which is expressed during the EBV lytic phase, makes tumor cells more susceptible to the antiviral drug ganciclovir [59]. The combination of a synthetic BZLF1-targeted transcriptional activator and ganciclovir has shown the highly selective cytotoxic effects of lytic induction therapy against EBV-positive tumor cells [57]. The CRISPR–Casilio Activator has been found to activate EBV lytic genes and exhibit cytotoxicity in NPC and EBV-associated GC cells [60]. Additionally, Aspirin depletes p65 in the nucleus and reactivates EBV into lytic replication, which reduces B lymphocyte viability [61]. To date, clinical trials have demonstrated promising outcomes by evaluating the effects of combining lytic inducers, such as gemcitabine and valproic acid, with valganciclovir in patients with end-stage NPC [62]. This study demonstrates that the treatment of resveratrol or/and cisplatin activates the EBV lytic cycle. This excessive lytic replication promotes cell rupture and inhibits tumor growth. Therefore, the combination of resveratrol and cisplatin might be broadly applicable to EBV-positive cancers.

So, our study not only reveals the anticancer activity of resveratrol in EBV-positive NPC and GC cells but also provides a potential approach to enhancing the sensitivity of cisplatin in these cells.

## 4. Materials and Methods

### 4.1. Cell Lines and Culture

Nasopharyngeal carcinoma cell lines, including HONE1, HONE1-EBV, HNE2, HNE2-LMP1, HK1 and HK1-EBV, were cultured in RPMI-1640 medium (Pricella, Wuhan, China) supplemented with 10% FBS (BI). The gastric cancer cell lines AGS and AGS-EBV were cultured in Ham’s F-12 (Pricella) supplemented with 10% FBS. All cells were maintained at 37 °C with 5% CO_2_. All cell lines were regularly tested for mycoplasma contamination by using a PCR-based assay (#A8994; AppliChem, Darmstadt, Germany), and all cell lines tested negative for mycoplasma contamination.

### 4.2. Reagents and Antibodies

The chemicals resveratrol (Cat: HY-16561, NJ, USA), acetylcysteine (Cat: HY-B0215, NJ, USA), cisplatin (Cat: HY-17394, NJ, USA), cocktail (Cat: HY-K0010, NJ, USA) and cycloheximide (Cat: HY-12320, NJ, USA) were purchased from MedChemExpress (Monmouth Junction, NJ, USA).

The antibodies used were rabbit anti-PTPN1 (Cat: HPA012542, Sigma, St. Louis, MO, USA) and mouse anti-β-actin (Cat: A5441, Sigma).

### 4.3. Cell Viability Assay

For the viability assay, 4 × 10^3^ cells per well were seeded into 96-well culture plates. The cells were then treated with varying concentrations of resveratrol for predetermined periods of time. The ratio of culture medium to Cell Counting Kit-8 (CCK-8, Beyotime, Shanghai, China) reagent was 9:1. A 100 μL volume of this mixture was then added to each well. The absorption at 450 nm was measured after incubating the plates for 60 min at 37 °C with 5% CO_2_. Following this incubation period, the plates were gently shaken to ensure even mixing, and color intensity was quantitatively assessed at a 450 nm wavelength using a BioTek 800 TS microplate reader (BioTek, Winooski, VT, USA).

### 4.4. Cell Count Assay

For the cell count assay, 4 × 10^3^ cells per well were seeded into 96-well culture plates. After 24 h of incubation with 80 µM resveratrol, the cells were counted by using a Lionheart LX automated microscope (BioTek, Winooski, VT, USA).

### 4.5. RNA Isolation and Real-Time Quantitative PCR (qPCR)

Total RNA was isolated utilizing the NucleoZOL reagent (Cat: 740404, MACHEREY-NAGEL GmbH & Co. KG, Düren, Germany) according to the manufacturer’s instructions. Briefly, cells were lysed directly in culture plates by adding 0.5 mL of NucleoZOL reagent per 6 cm^2^ culture area. After homogenization, 0.2 mL of nuclease-free water was added per 0.5 mL of NucleoZOL, and samples were incubated at room temperature for 15 min. Phase separation was achieved by centrifugation at 12,000× *g* for 15 min at 4 °C, and RNA was precipitated from the aqueous phase with an equal volume of isopropanol. RNA pellets were washed twice with 75% ethanol, air-dried, and dissolved in nuclease-free water. RNA concentration and purity were determined using a NanoDrop 2000 spectrophotometer (Thermo Fisher Scientific, Waltham, MA, USA), with A260/A280 ratios between 1.8 and 2.0 considered acceptable. cDNA synthesis from the harvested mRNA was conducted using the RevertAid First Strand cDNA Synthesis Kit (Cat: K1622, Invitrogen, Carlsbad, CA, USA) in accordance with its protocol for reverse transcription. Real-time quantitative PCR was carried out in triplicate, employing SYBR™ Green Master Mix (Cat: A25742, Invitrogen) on the ABI7500 Real-Time PCR System (Applied Biosystems, San Francisco, CA, USA). Each 20 μL reaction contained 10 μL of SYBR Green Master Mix, 0.5 μM each of forward and reverse primers, and 5 μL of diluted cDNA. Relative gene expression was calculated using the ΔΔCt method, with GAPDH as the internal reference gene.

The primer sequences applied in this investigation are listed below:

BZLF1 forward: 5′-CATGTTTCAACCGCTCCGACTGG-3′;
BZLF1 reverse: 5′-GCGCAGCCTGTCATTTTCAGATG-3′;
BMRF1 forward: 5′-CTAGCCGTCCTGTCCAAGTGC-3′;
BMRF1 reverse: 5′-AGCCAAACGCTCCTTGCCCA-3′;
BALF2 forward: 5′-CCCCGGGACTTTATCAAGATGTTC-3′;
BALF2 reverse: 5′-CCACCTCGGCGTCCAC-3′;
IFN-α4 forward: 5′-AACCTAGAGGCCGAAGTTCAAG-3′;
IFN-α4 reverse: 5′-TGTGGGTCTGAGGCAGATCA-3′;
IFN-β1 forward: 5′-ACACTGGTCGTGTTGTTGAC-3′;
IFN-β1 reverse: 5′-GGAAAGAGCTGTCGTGGAGA-3′;
IL-6 forward: 5′-CAGCCCTGAGAAAGGAGACAT-3′;
IL-6 reverse: 5′-GGTTCAGGTTGTTTTCTGCCA-3′;
GAPDH forward: 5′-GACATCAAGAAGGTGGTGAA-3′;
GAPDH reverse: 5′-TGTCATACCAGGAAATGAGC-3′.

### 4.6. Western Blot Analysis

After treatment with resveratrol, cells were rinsed with PBS and subsequently lysed on ice for 1 h in RIPA buffer containing a protease inhibitor cocktail. Lysates were then centrifuged at 13,000 rpm for 15 min at 4 °C, and supernatants were collected. Protein concentrations were determined using the BCA assay. Next, 30 μg of protein per sample was resolved by 10% SDS-PAGE and electro-transferred to PVDF membranes (Millipore, Burlington, MA, USA). Membranes were blocked with 5% non-fat milk in PBS for 1 h at room temperature. Following blocking, membranes were incubated overnight at 4 °C with primary antibodies diluted in PBS. After three washes with PBST, membranes were incubated with HRP-conjugated anti-mouse or anti-rabbit secondary antibodies for 1 h at room temperature. Bands were visualized using the ChemiDoc^TM^ XRS+ system with Image Lab^TM^ v5.0 software (Bio-Rad, Hercules, CA, USA). And protein expression levels were normalized to β-actin.

### 4.7. EdU Assay

Proliferating cells were stained using the EdU Cell Proliferation Kit with Alexa Fluor 647 from BeyoClick (Cat: C0081L, Beyotime, Shanghai, China), following the manufacturer’s instructions. Cells were seeded in 96-well culture plates and treated with resveratrol for 24 h. The EdU working solution was prepared with fresh complete RPMI-1640 medium at a concentration of 20 mM. The 2X EdU working solution was preheated to 37 °C and added to the 96-well plate in equal volume, incubating at 37 °C, 5% CO_2_ for 3 h. Subsequently, cells were fixed with 4% paraformaldehyde for 20 min and permeabilized with 0.3% Triton X-100 for another 15 min at room temperature. Each well was then incubated with 60 μL of click additive solution for 30 min at room temperature in the dark. Cell nuclei were stained with Hoechst 33342 for 10 min for visualization. Fluorescence was quantified using the Lionheart LX automated microscope (BioTek, USA). The ratio of EdU-positive cells to the total number of cells represents the percentage of EdU-positive cells.

### 4.8. Apoptosis Assay

Cell apoptosis was determined by flow cytometric analysis. After 24 h of resveratrol incubation, cells were harvested and washed twice with ice-cold PBS according to the Annexin V-APC/7-AAD apoptosis detection kit instructions (BD Biosciences, Franklin Lakes, NJ, USA). Cells were then resuspended in 500 μL of binding buffer. Subsequently, 5 μL of Annexin V-APC and 10 μL of 7-AAD staining solution were added to the cell suspension. The mixture was incubated in the dark at room temperature for 5 min before conducting the flow cytometric evaluation.

### 4.9. Cellular Thermal Shift Assay (CETSA)

CETSA was executed in accordance with the method previously described in the literature [63]. In summary, cells were subjected to treatment with resveratrol or DMSO, followed by a 24 h incubation period. Post-treatment, the cell populations were gathered, washed with PBS, and subsequently resuspended to attain a cellular concentration of 5 × 10^6^ cells/mL in PBS that was enhanced with protease inhibitors. Subsequently, 50 μL aliquots of the cell suspension were allocated into PCR tubes. These aliquots were then exposed to thermal conditions ranging from 35 to 50 °C in a thermal cycle for a duration of 3 min, followed by immediate lysis through a freeze–thaw cycle in liquid nitrogen. The resulting cell lysates were then subjected to centrifugation at 13,000 rpm for 15 min at a chilled temperature of 4 °C. The acquired supernatants were finally submitted for immunoblotting analysis.

### 4.10. Ligand-Based Prediction Approach

In order to rapidly identify the potential target proteins of resveratrol, we initially aggregated the results from three widely used target prediction platforms: ChEMBL, TCMSP, and Swiss Target Prediction. We then cross-referenced the predicted targets from each platform to identify any overlapping proteins.

To validate these potential target proteins, we conducted functional analyses utilizing the Gene Ontology (GO) and Kyoto Encyclopedia of Genomes (KEGG) databases within the context of the Diversity Visualisation Integrated Database. We focused our attention on the GO terms and KEGG pathways that indicated statistical significance with *p* values of less than 0.05. This was done to ascertain whether the validated target proteins were principally associated with the known pharmacological actions of resveratrol.

### 4.11. Molecular Docking

To verify the target proteins screened previously, molecular docking was conducted to simulate the binding affinity between resveratrol and the target proteins with three-dimensional crystal structures. The principle of molecular docking involves calculating optimal binding geometries and energies by positioning the ligand in various orientations and conformations within the binding site. Initially, the X-ray crystal structures of the screened target proteins were downloaded from the Protein Data Bank (PDB) and prepared using AutoDockTools v4.2. This preparation included removing crystallographic water molecules and ions, adding fixed hydrogens, and assigning protonation states. The native ligand from each crystal structure was utilized to define the binding site. Molecular docking with the induced fit mode was then performed to investigate the interaction between resveratrol and the target proteins. The optimal structural conformation was determined based on the score values obtained.

### 4.12. Tumor Xenograft Studies

This animal research project was approved by the Institutional Animal Care and Use Committee (IACUC) of Central South University (No. 2020sydw0277). Female BALB/c-nude mice, aged 4–5 weeks, were acquired from Vital River Laboratory Animal Technology Co., Ltd. (Beijing, China). All mice were subcutaneously inoculated with HONE1-EBV cells (5 × 10^6^ cells/mouse) in the right armpit. When the tumors reached a volume of 100 mm^3^ on the Fourth day post-injection, the mice were randomly allocated into four groups (n = 6 per group): saline treatment (control), resveratrol treatment, cisplatin treatment, and combination treatment with resveratrol and cisplatin. Treatment commenced with either resveratrol (30 mg/kg) or cisplatin (4 mg/kg) through intraperitoneal injection. Control animals received 0.9% saline as the vehicle. Tumor volume and body weight were measured daily. Tumor size was determined using the formula tumor size = (π × length × width^2^)/6. After 8 d of treatment, the mice were humanely euthanized, and tumors were excised and weighed.

### 4.13. Immunohistochemistry (IHC)

Tumor tissue sections were deparaffinized using an environmentally friendly dewaxing agent (Solarbio, Beijing, China) and subsequently rehydrated with a series of ethanol aqueous solutions in decreasing concentrations. For antigen retrieval, the tissue sections were incubated in 10 mM sodium citrate buffer for 10 min in a microwave oven set to the highest power. Endogenous peroxidase activity was blocked by incubating the sections with 3% hydrogen peroxide for 10 min, followed by blocking with normal donkey serum for 30 min. The primary antibodies were applied overnight at 4 °C. Chromogen development was performed using DAB (Zsgbbio, Beijing, China), and the sections were counterstained with a hematoxylin staining kit.

### 4.14. Statistical Analysis

All statistical analyses were performed using GraphPad Prism 8 software. Data are presented as the mean ± standard error of the mean (SEM) from at least three independent experiments (n ≥ 3). Each independent experiment was performed with triplicate or quintuplicate technical replicates, as specified in the figure legends. Comparisons between two groups were analyzed using two-tailed Student’s *t*-test. Levels of *p* < 0.05 were considered statistically significant (* *p* < 0.05, ** *p* < 0.01, *** *p* < 0.001; NS, no significance).

## 5. Conclusions

In this study, we employed an integrated strategy combining target prediction, molecular docking, and experimental validation to investigate the anticancer mechanisms of resveratrol in EBV-positive nasopharyngeal and gastric carcinomas. Our results demonstrate that resveratrol exerts potent cytotoxic effects against EBV-positive cancer cells by directly targeting PTPN1, a non-receptor protein tyrosine phosphatase overexpressed in these malignancies. We further showed that resveratrol promotes PTPN1 degradation via the proteasome pathway, thereby impairing cell viability and enhancing the sensitivity of EBV-positive cells to cisplatin. Moreover, resveratrol alone or in combination with cisplatin induces EBV lytic reactivation, contributing to its anti-tumor activity.

This study reveals a previously unrecognized anticancer mechanism of resveratrol. By simultaneously targeting the host resistance factor PTPN1 and inducing the cytotoxic EBV lytic cycle, this dual-pronged mechanism offers a distinct therapeutic advantage over conventional agents. Given resveratrol’s well-documented safety profile, it holds promise as an ideal adjuvant for combination therapy. The synergistic interaction between resveratrol and cisplatin provides a rational combination strategy to overcome cisplatin resistance, which is particularly relevant for patients with advanced EBV-associated tumors who have failed standard chemotherapy. These findings further establish PTPN1 as a promising therapeutic target.

These findings elucidate a novel molecular mechanism underlying resveratrol’s anticancer effects and reinforce PTPN1 as a compelling therapeutic target in EBV-associated cancers. The synergy observed between resveratrol and cisplatin provides a rational basis for combination strategies aimed at overcoming cisplatin resistance. Overall, this work supports the further development of resveratrol as an adjuvant agent for the treatment of EBV-positive malignancies and offers a translational framework for targeting PTPN1 in combination with conventional chemotherapy. Future studies should focus on validating the clinical efficacy of this combination strategy in patient-derived xenograft models and exploring the broader applicability of PTPN1-targeted therapy across other EBV-associated malignancies.

## Figures and Tables

**Table 1:** The IC50 of resveratrol on HONE1-EBV, HK1-EBV, HNE2-LMP1 and AGS-EBV cells.

Cell/IC50 (μM)	24 h	48 h	72 h
HONE1-EBV	133.9	104.5	67.45
HK1-EBV	82.18	73.63	58.13
HNE2-LMP1	145.7	112.9	80.17
AGS-EBV	88.78	60.89	35.85

**Table 2:** Univariate and multivariate COX regression analyses of overall survival of HNSCC patients.

Variable	Univariate Analysis			Multivariate Analysis		
	HR	95% CI	*p* Value	HR	95% CI	*p* Value
Age (≥61 vs. <61)	1.29	0.97–1.69	0.0751			
Sex (Male vs. Female)	0.72	0.54–0.97	0.0325 *			
Stage (IV vs. I/II/III)	1.76	1.28–2.43	5.53 × 10^−4^ *	1.92	1.38–2.66	9.02 × 10^−5^ *
pT (T3/T4 vs. T1/T2)	1.68	1.27–2.24	3.5 × 10^−4^ *	1.68	1.26–2.24	3.58 × 10^−4^ *
pN (N1/N2/N3 vs. N0)	1.62	1.23–2.13	6.05 × 10^−4^ *	1.76	1.33–2.32	7.3 × 10^−5^ *
PTPN1 expression (High vs. Low)	1.34	1.02–1.77	0.037 *	1.37	1.04–1.81	0.0253 *

*, significant difference.

## Data Availability

The original contributions presented in this study are included in the article/Supplementary Materials. Further inquiries can be directed to the corresponding authors.

## Supplementary Materials

The following supporting information can be downloaded at https://www.mdpi.com/article/10.3390/ph19040603/s1, Figure S1: (**A**) The EdU staining was performed to assess the S-phase cell cycle progression in cells treated with DMSO and resveratrol (80 µM; RES: Resveratrol). Hoechst 33342 staining was used to visualize the nuclei. (**B**) Flow cytometry analysis of cell death in EBV/LMP1-positive NPC and GC cells treated with resveratrol (80 µM) for 24 h; Figure S2: (**A**) Flow cytometry analysis of ROS in EBV/LMP1-positive NPC and GC cells treated with resveratrol (80 µM) for 24 h (n = 3; ***, *p* < 0.001). (**B**) NPC and GC cells were treated with NAC (5 mM), resveratrol (80 µM), or a combination of both NAC (5 mM) and resveratrol (80 µM) for 24 h. Cell viability was then assessed using the CCK-8 assay (n = 5; NS, >0.05; *, *p* < 0.05; **, *p* < 0.01; ***, *p* < 0.001); Figure S3: (**A**) Comparative analysis of MMP2, BCL2, CA2, and PTGS2 expression in HNSCC and GC patients from TCGA database (TCGA-HNSCC dataset: Normal = 44, Tumor = 520; TCGA-GC dataset: Normal = 36, Tumor = 414; NS, >0.05; *, *p* < 0.05; **, *p* < 0.01; ***, *p* < 0.001). (**B**) Analysis of MMP2, BCL2, CA2, and PTGS2 expression using the GEO database (GSE178537: Head and neck squamous cell carcinoma samples, Normal = 21, Tumor = 44; GSE66229: Gastric cancer samples, Normal = 100, Tumor = 300; NS, >0.05; **, *p* < 0.01; ***, *p* < 0.001). (**C**) Kaplan–Meier curve showing the overall survival probability of HNSCC patients from the TCGA database with low or high mRNA levels of MMP9. (**D**) The optimal structural conformation of PTPN1 (Purple) and the ligand resveratrol (Pink). docking score= −4.89; Figure S4: (**A**) EBV/LMP1-positive NPC and GC cells treated with resveratrol (80 μM) for 24 h were analyzed by qPCR to detect mRNA expression levels of IFN-α, IFN-β, and IL-6 (n = 3, *, *p* < 0.05; **, *p* < 0.01; ***, *p* < 0.001). (**B**) The mouse weight curve of the different treatment groups (n = 5); Table S1: Fifty-eight potential medicinal targets.
