# Supplementary material for: Resveratrol Induces Proteasomal Degradation of PTPN1 to Enhance Cisplatin Sensitivity in Epstein–Barr Virus-Associated Malignancies
Source: Pharmaceuticals (Basel). 2026 Apr 9;19(4):603. doi: 10.3390/ph19040603 (PMC13118724; doi:10.3390/ph19040603)
Supplement: Supplementary file 1 [file pharmaceuticals-19-00603-s001.zip › pharmaceuticals-4231366-supplementary.pdf]

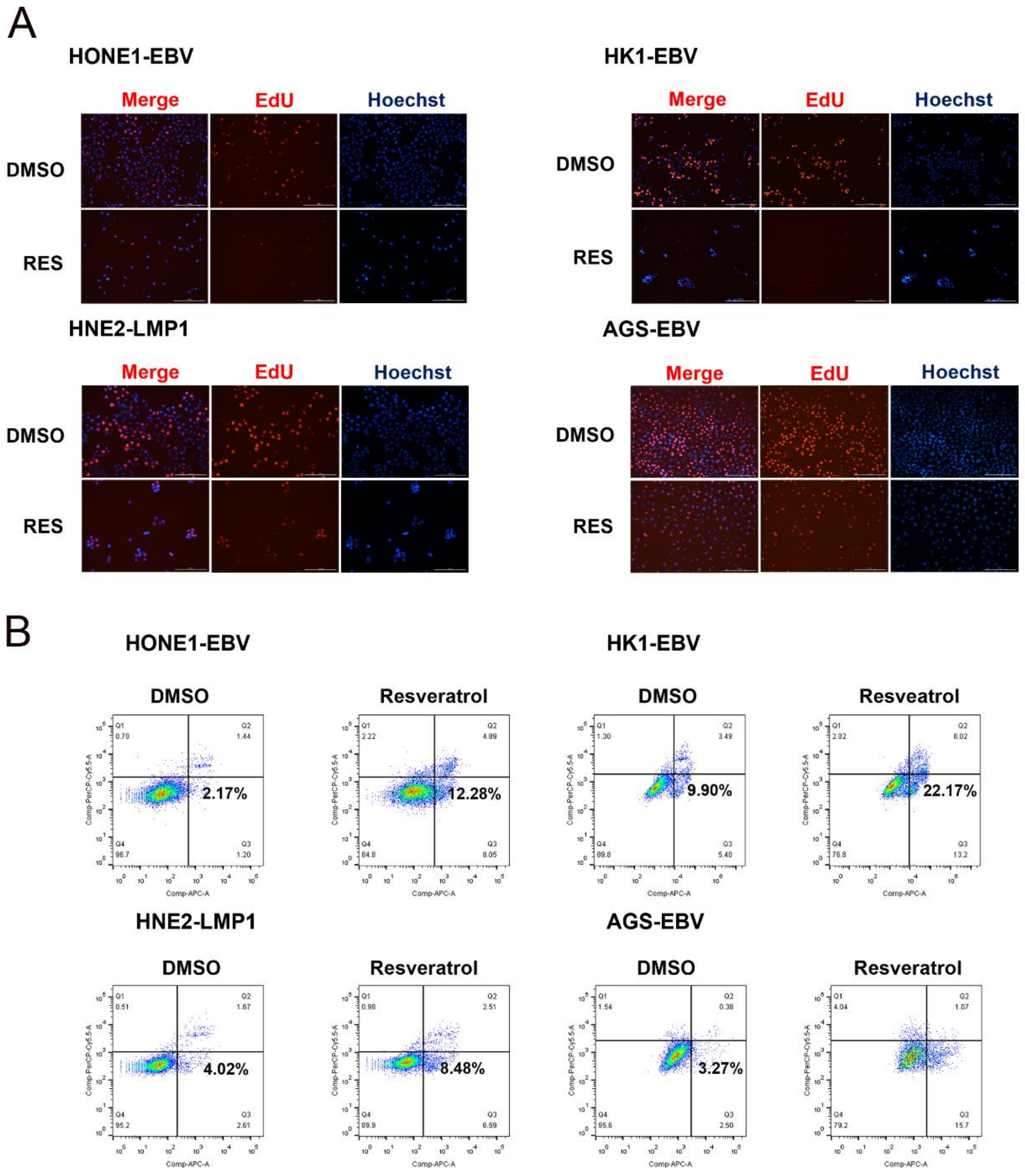

Supplementary Figure S1. (**A**) The EdU staining was performed to assess the S-phase cell cycle progression in cells treated with DMSO and resveratrol (80 μM; RES: Resveratrol). Hoechst 33342 staining was used to visualize the nuclei. (**B**) Flow cytometry analysis of cell death in EBV/LMP1-positive NPC and GC cells treated with resveratrol (80 μM) for 24 hours.

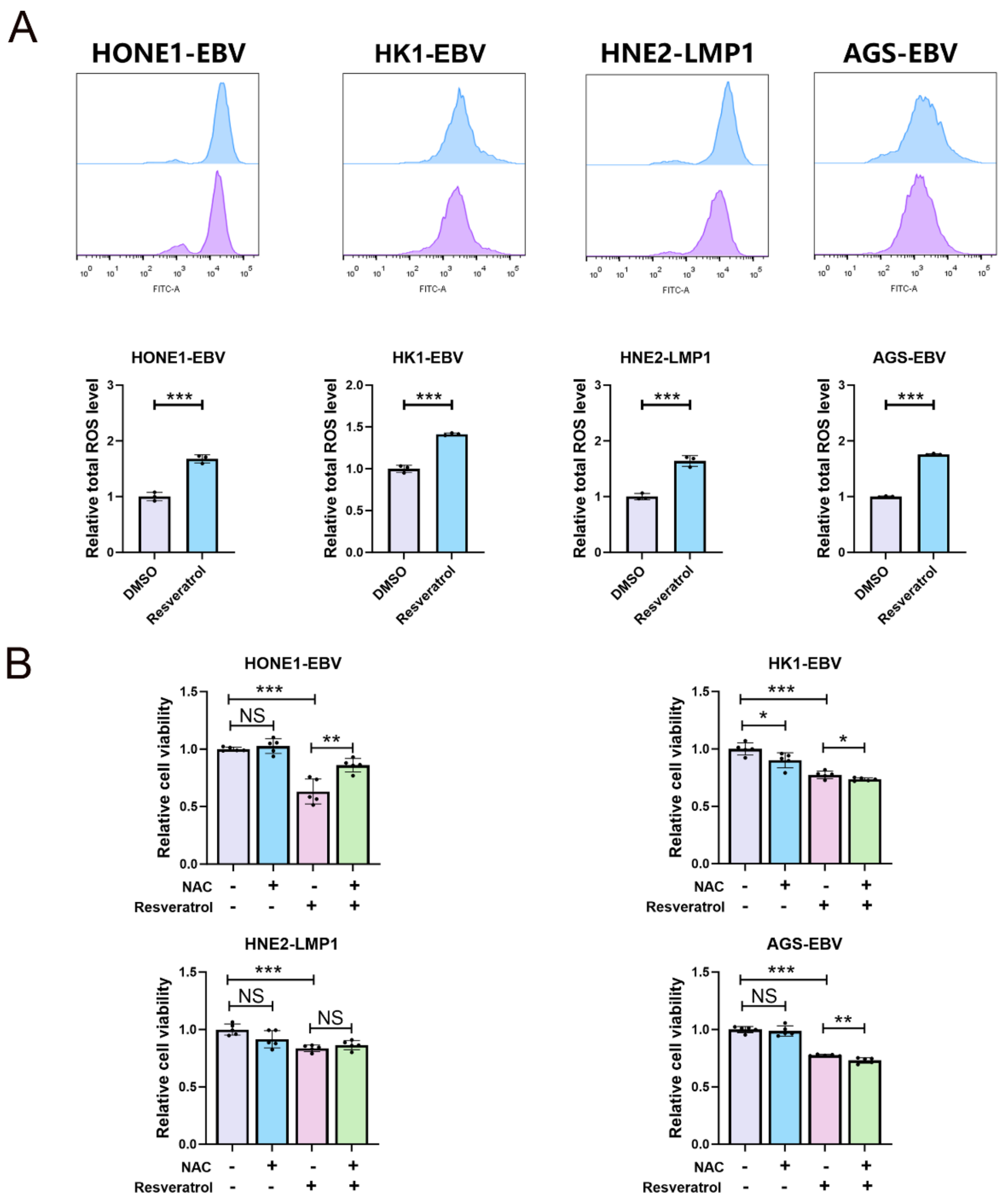

Supplementary Figure S2. (**A**) Flow cytometry analysis of ROS in EBV/LMP1-positive NPC and GC cells treated with resveratrol (80 μM) for 24 hours (n=3; ***, $P < 0.001$). (**B**) NPC and GC cells were treated with NAC (5 mM), resveratrol (80 μM), or a combination of both NAC (5 mM) and resveratrol (80 μM) for 24 hours. Cell viability was then assessed using the CCK-8 assay (n=5; NS, > 0.05; *, $P < 0.05$; **, $P < 0.01$; ***, $P < 0.001$).

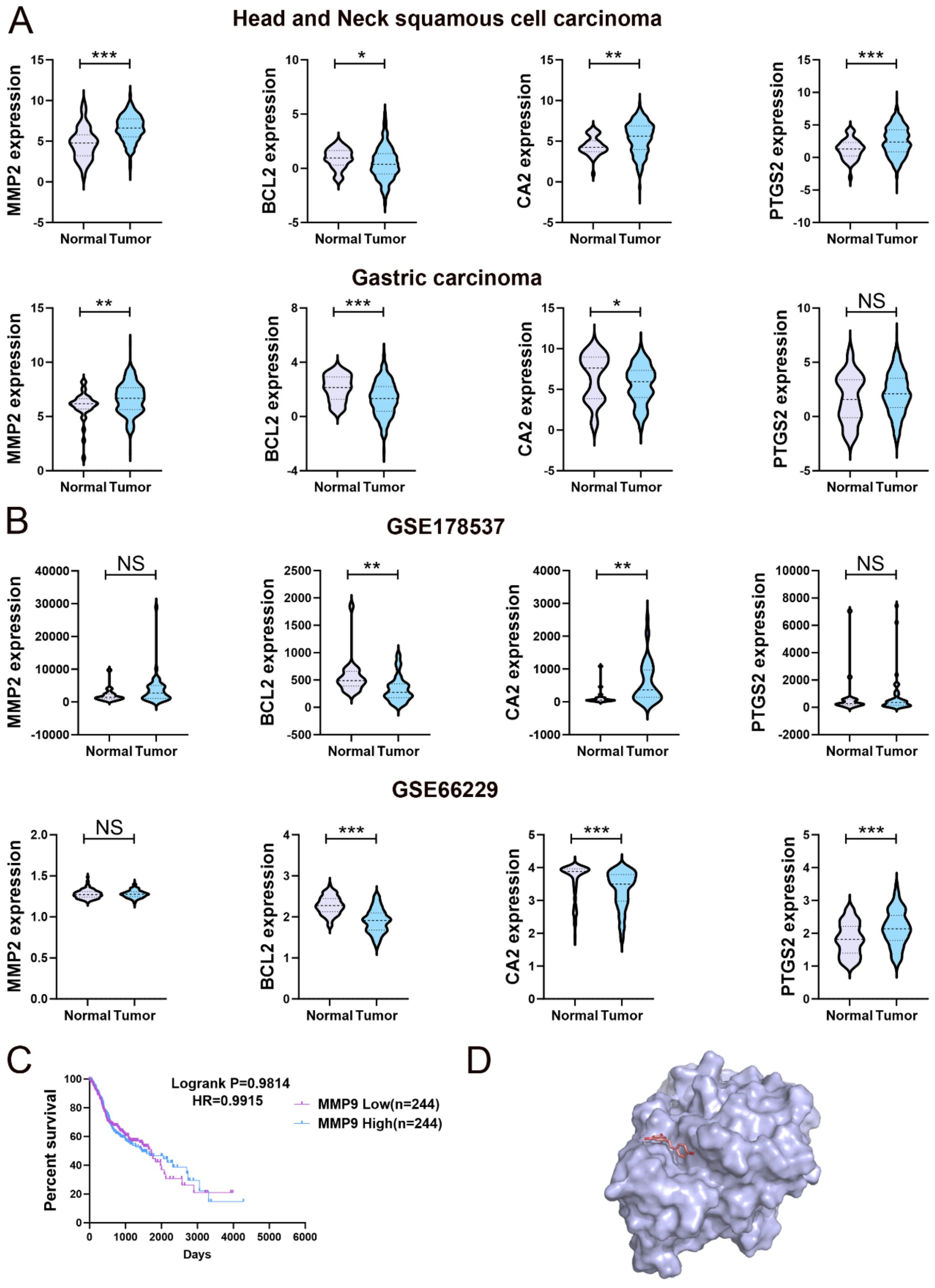

Supplementary Figure S3. (**A**) Comparative analysis of MMP2, BCL2, CA2, and PTGS2 expression in HNSCC and GC patients from TCGA database (TCGA-HNSCC dataset: Normal=44, Tumor=520; TCGA-GC dataset: Normal=36, Tumor=414; NS,   > 0.05; *, P < 0.05; **, P < 0.01; ***, P < 0.001). (**B**) Analysis of MMP2, BCL2, CA2, and PTGS2 expression using the GEO database (GSE178537: Head and neck squamous cell carcinoma samples, Normal=21, Tumor=44; GSE66229: Gastric cancer samples, Normal=100, Tumor=300; NS,   > 0.05; **, P < 0.01; ***, P < 0.001). (**C**) Kaplan–Meier curve showing the overall survival probability of HNSCC patients from the TCGA database

with low or high mRNA levels of MMP9. (**D**) The optimal structural conformation of PTPN1 (Purple) and the ligand resveratrol (Pink). docking score= -4.89.

A

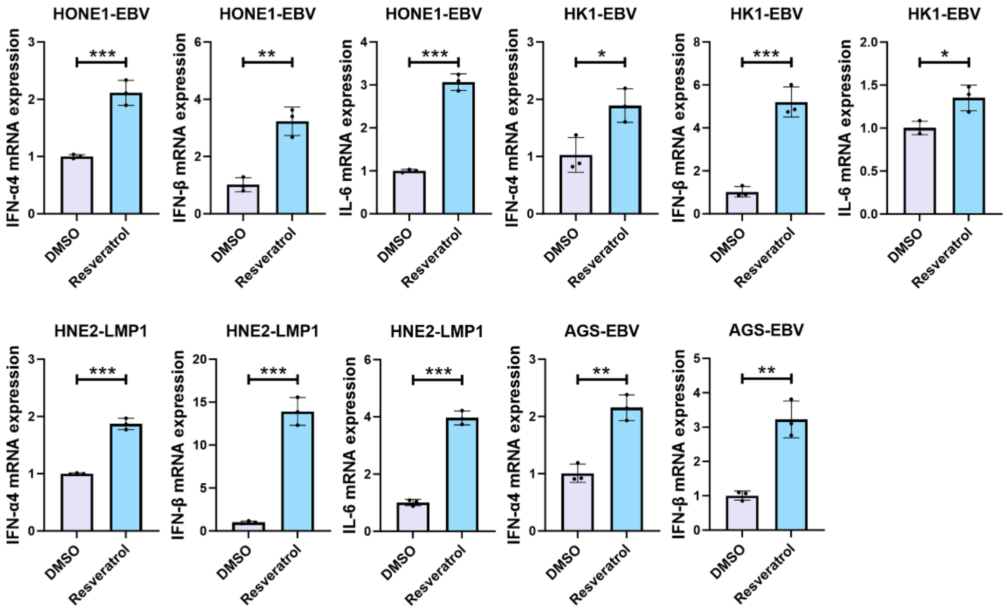

B

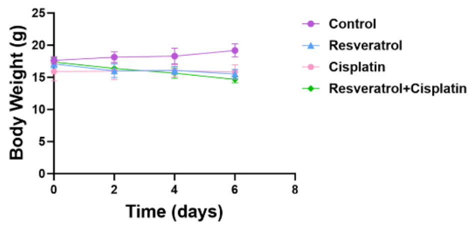

Supplementary Figure S4. (**A**) EBV/LMP1-positive NPC and GC cells treated with resveratrol (80 μM) for 24 hours were analyzed by qPCR to detect mRNA expression levels of IFN-α, IFN-β, and IL-6 (n=3, *, $P < 0.05$; **, $P < 0.01$; ***, $P < 0.001$). (**B**) The mouse weight curve of the different treatment groups (n=5).

Supplementary Table S1 Fifty-eight potential medicinal targets

Gene	Proteins
**MMP2**	Matrix metallopeptidase 2
**PTPN1**	Protein tyrosine phosphatase non-receptor type 1
**PTGS2**	Prostaglandin-endoperoxide synthase 2
**BCL2**	BCL2 apoptosis regulator
**CA2**	Carbonic anhydrase 2
**MMP9**	Matrix metallopeptidase 9
**SLC6A2**	Solute carrier family 6 member 2
**CA13**	Carbonic anhydrase 13
**LCK**	LCK proto-oncogene
**RAF1**	Raf-1 proto-oncogene
**DRD2**	Dopamine receptor D2
**CA5B**	Carbonic anhydrase 5B
**CYP1A2**	Cytochrome P450 family 1 subfamily A member 2
**CA14**	Carbonic anhydrase 14
**CA9**	Carbonic anhydrase 9
**ABCC1**	ATP binding cassette subfamily C member 1
**NOX4**	NADPH oxidase 4
**ESR1**	Estrogen receptor 1
**SHBG**	Sex hormone binding globulin
**F10**	Coagulation factor X
**ESRRA**	Estrogen related receptor alpha
**BCL2L1**	BCL2 like 1
**AKR1B1**	Aldo-keto reductase family 1 member B
**ESR2**	Estrogen receptor 2
**VCP**	Valosin containing protein
**NQO2**	N-ribosyldihydronicotinamide: quinone dehydrogenase 2
**MMP1**	Matrix metallopeptidase 1
**CA12**	Carbonic anhydrase 12
**APP**	Amyloid beta precursor protein
**CDK2**	Cyclin dependent kinase 2
**CA7**	Carbonic anhydrase 7
**XDH**	Xanthine dehydrogenase
**TP53**	Tumor protein p53
**TNF**	Tumor necrosis factor

**SIRT2**	Sirtuin 2
**CCR2**	C-C motif chemokine receptor 2
**XIAP**	X-linked inhibitor of apoptosis
**NQO1**	NAD(P)H quinone dehydrogenase 1
**CCND1**	Cyclin D1
**CDK4**	Cyclin dependent kinase 4
**PLAUR**	Plasminogen activator
**PPARG**	Peroxisome proliferator activated receptor gamma
**MCL1**	MCL1 apoptosis regulator
**PRKCA**	Protein kinase C alpha
**CASP3**	Caspase 3
**HSP90AA1**	Heat shock protein 90 alpha family class A member 1
**MTOR**	Mechanistic target of rapamycin kinase
**CYP19A1**	Cytochrome P450 family 19 subfamily A member 1
**PLAT**	Plasminogen activator
**CCNE1**	Cyclin E1
**PTGS1**	Prostaglandin-endoperoxide synthase 1
**F3**	Coagulation factor III
**IGF1R**	Insulin like growth factor 1 receptor
**CYP1A1**	Cytochrome P450 family 1 subfamily A member 1
**RELA**	NF-kB subunit
**NOS3**	Nitric oxide synthase 3
**CYP1B1**	Cytochrome P450 family 1 subfamily B member 1
**AHR**	Aryl hydrocarbon receptor
